# The promising role of noncoding RNAs in cancer-associated fibroblasts: an overview of current status and future perspectives

**DOI:** 10.1186/s13045-020-00988-x

**Published:** 2020-11-19

**Authors:** Zengli Fang, Jin Xu, Bo Zhang, Wei Wang, Jiang Liu, Chen Liang, Jie Hua, Qingcai Meng, Xianjun Yu, Si Shi

**Affiliations:** 1 Department of Pancreatic Surgery, Fudan University Shanghai Cancer Center, No. 270 Dong’An Road, Shanghai, 200032 China; 2 Department of Oncology, Shanghai Medical College, Fudan University, Shanghai, 200032 China; 3 Shanghai Pancreatic Cancer Institute, Shanghai, 200032 China; 4 Pancreatic Cancer Institute, Fudan University, Shanghai, 200032 China

**Keywords:** Noncoding RNA, MicroRNA, Long noncoding RNA, Cancer-associated fibroblasts, Tumor microenvironment, Cancer, Exosome, Cell–cell interaction

## Abstract

As the most important component of the stromal cell population in the tumor microenvironment (TME), cancer-associated fibroblasts (CAFs) are crucial players in tumor initiation and progression. The interaction between CAFs and tumor cells, as well as the resulting effect, is much greater than initially expected. Numerous studies have shown that noncoding RNAs (ncRNAs) play an irreplaceable role in this interplay, and related evidence continues to emerge and advance. Under the action of ncRNAs, normal fibroblasts are directly or indirectly activated into CAFs, and their metabolic characteristics are changed; thus, CAFs can more effectively promote tumor progression. Moreover, via ncRNAs, activated CAFs can affect the gene expression and secretory characteristics of cells, alter the TME and enhance malignant biological processes in tumor cells to contribute to tumor promotion. Previously, ncRNA dysregulation was considered the main mechanism by which ncRNAs participate in the crosstalk between CAFs and tumor cells. Recently, however, exosomes containing ncRNAs have been identified as another vital mode of interaction between these two types of cells, with a more direct and clear function. Gaining an in-depth understanding of ncRNAs in CAFs and the complex regulatory network connecting CAFs with tumor cells might help us to establish more effective and safer approaches for cancer therapies targeting ncRNAs and CAFs and offer new hope for cancer patients.

## Introduction

Tumor progression is not an independent event related only to the biological characteristics of tumor cells but instead is a process affected and modulated by many factors. One of these factors, the tumor microenvironment (TME), is vital for tumor initiation, progression, metastasis, immunosuppression and therapeutic resistance [1, 2]. The TME consists of cancer-associated fibroblasts (CAFs), macrophages, myeloid-derived suppressor cells (MDSCs), lymphocytes, antigen-presenting cells and other tumor-associated stromal cells, as well as microvessels, lymphatic vessels, nerves, infiltrating biomolecules and extracellular matrix (ECM) components [3, 4]. As the key components among these constituents, CAFs are abundant in the TME (especially in pancreatic cancer) and interact closely with tumor cells [5]. Fibroblasts are a population of spindle-shaped cells of mesenchymal origin that exhibit distinct phenotypes according to the microenvironment. Fibroblasts activated during wound healing are called “myofibroblasts,” while those activated in tumor tissues are defined as CAFs [6, 7]. Unlike normal fibroblasts (NFs), most CAFs have a myofibroblastic phenotype (termed myCAFs) with characteristics such as overexpression of α-smooth muscle actin (α-SMA) and fibroblast activation protein (FAP) and are in a state of active proliferation and metabolism [8]. Additionally, some CAFs are defined as inflammatory CAFs (iCAFs) due to their secretory properties and inflammatory regulatory function [9]. CAFs contribute substantially to tumor progression in multifarious manners. As the main source of collagen-producing cells in the TME, CAFs provide mechanical support for tumor tissues and regulate the growth and invasion of tumor cells by synthesizing or remodeling the structure of the ECM [10]. Importantly, CAFs can also mediate the interplay between tumor cells and stromal cells by secreting a variety of growth factors, cytokines, chemokines and exosomes, thus affecting the key steps in tumor malignant progression [11, 12]. However, the specific mechanisms through which CAFs play these roles need further exploration.

Only < 2% of the transcripts in the human genome encode proteins; the remaining 98% are noncoding RNAs (ncRNAs). Because they lack complete open reading frames (ORFs), ncRNAs cannot be templates for protein synthesis, but they contribute significantly to the regulation of epigenetic modification levels. NcRNAs are classified into two main categories according to their length: small noncoding RNAs (sncRNAs) and long noncoding RNAs (lncRNAs). SncRNAs are usually less than 200 nucleotides (nt) in length and include microRNAs (miRNAs), piwi-interacting RNAs (piRNAs), small interfering RNAs (siRNAs), and so on. MiRNAs are a class of sncRNAs with a length of approximately 22 nt that mediate inhibition of gene translation or degradation of mRNA by binding to the 3′ untranslated region (3′ UTR) or ORFs of their target mRNA, thereby regulating the expression of target proteins at the posttranscriptional level [13]. NcRNAs with a transcript length of more than 200 nt are defined as lncRNAs; lncRNAs regulate gene expression at the transcriptional or posttranscriptional level through interactions with proteins or nucleic acids, thus affecting many biological processes [14]. Circular RNAs (circRNAs) are a new class of endogenous ncRNAs with a single-stranded, closed-loop structure that lacks 5′ and 3′ ends and a poly(A) tail, which makes circRNAs more stable than linear RNAs [15]. CircRNAs act as miRNA sponges or transcriptional regulators or bind to RNA-binding proteins (RBPs) to regulate gene expression [16]. NcRNAs participate in many physiological and pathological processes in the body, and ncRNA dysregulation is closely related to the development of diseases such as cardiovascular diseases, neurological diseases and cancer [17–20]. During tumor progression, ncRNAs can regulate the proliferation, stemness, metabolism, differentiation, apoptosis, invasion and drug resistance of tumor cells, while their dysregulation can be affected by gene mutations or epigenetic changes in tumor cells [21–23]. More importantly, ncRNAs can mediate the interplay between tumor cells and stromal cells via various mechanisms to promote or suppress tumor progression.

The role of ncRNAs in tumor cells has been confirmed by extensive research, and their effects on the TME have received increasing attention. Accumulating evidence shows that miRNAs are involved in the transformation of NFs to CAFs and modulate the crosstalk between CAFs and tumor cells. Although the research on the role of lncRNAs in CAFs is not as extensive and in-depth as that on the role of miRNAs, it has achieved remarkable advances. Importantly, tumor cells and CAFs can communicate more directly and effectively by producing exosomes packed with ncRNAs, which can be taken up by adjacent or distant cells to further contribute to tumor progression. To date, no study has investigated the function of other types of ncRNAs, such as circRNAs, in CAFs. Thus, this review focuses on the studies of miRNAs and lncRNAs. In this review, we introduce the origins and heterogeneity of CAFs; focus on the roles of miRNAs and lncRNAs in the formation and metabolic reprogramming of CAFs as well as on the contribution of miRNAs and lncRNAs in CAFs to aspects of tumor progression, such as proliferation, stemness, angiogenesis, metastasis and therapeutic resistance; and discuss the possible therapeutic targets among these mechanisms.

## Origins of CAFs

**Fig. 1:**
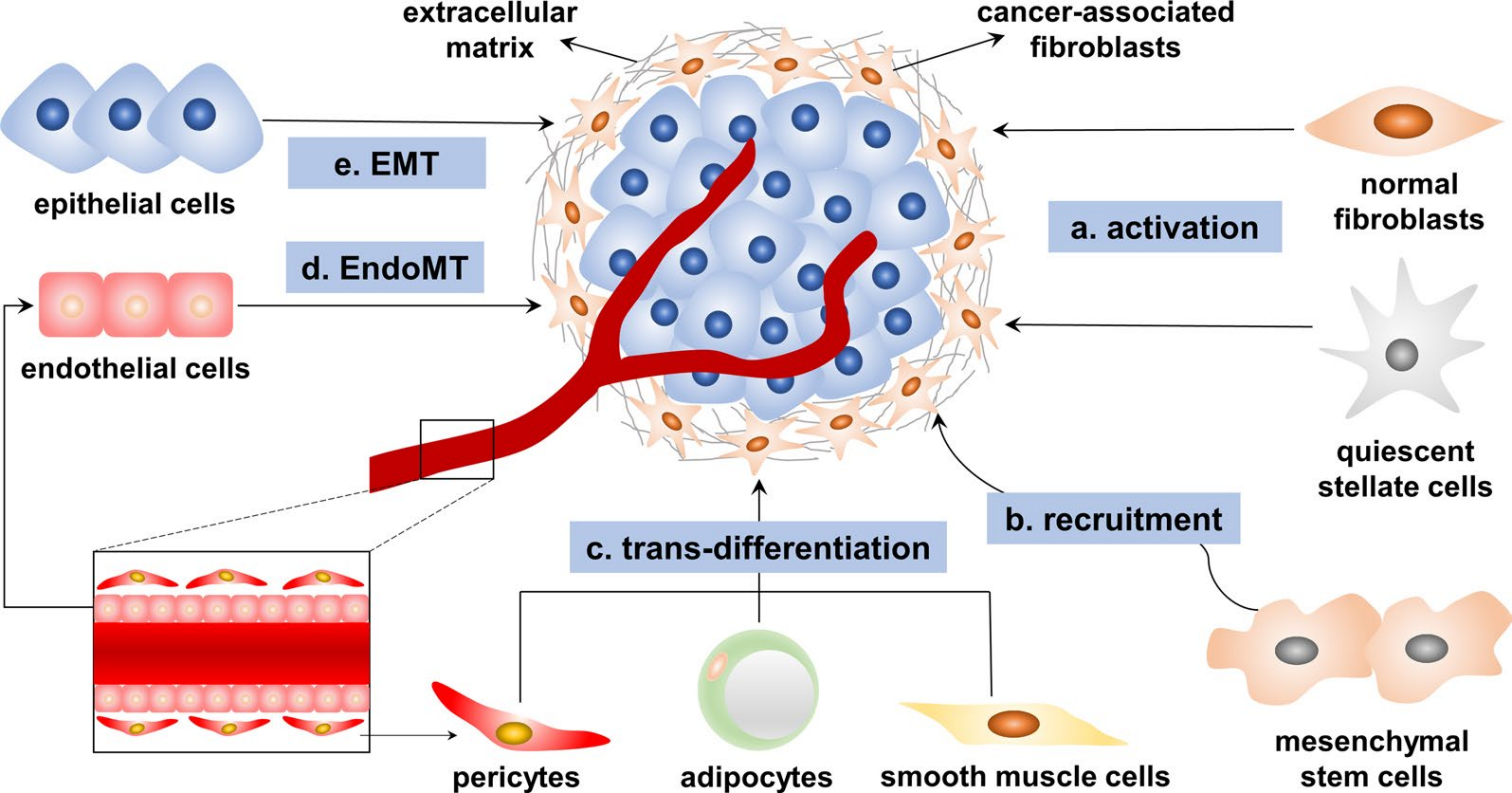
The origins of cancer-associated fibroblasts (CAFs). CAFs are a population of heterogeneous cells of multiple origins that originate mainly via the following mechanisms: **a** tissue-resident normal fibroblasts and quiescent stellate cells are activated into CAFs when stimulated by factors such as transforming growth factor-β (TGF-β), epidermal growth factor (EGF), platelet-derived growth factor (PDGF), fibroblast growth factor 2 (FGF2), sonic hedgehog (SHH), bone morphogenetic protein (BMP) and reactive oxygen species (ROS); **b** mesenchymal stem cells are recruited by cytokines and chemokines secreted into the tumor microenvironment, such as TGF-β, CXCR6, CXCL16, CXCL12, CCL2 and CCL5; **c** other cells such as pericytes, adipocytes and smooth muscle cells are transdifferentiated into CAFs; **d** endothelial cells are transformed into CAFs via endothelial-to-mesenchymal transition (EndoMT); and **e** epithelial cells are transformed into CAFs via epithelial-to-mesenchymal transition (EMT)

CAFs are a heterogeneous population of cells, and their heterogeneity might be attributable to their numerous potential cellular precursors (Fig. 1) [6, 9]. Tissue-resident NFs can be activated as CAFs under the stimulatory effects of the TME. Transforming growth factor-β (TGF-β), epidermal growth factor (EGF), platelet-derived growth factor (PDGF), fibroblast growth factor 2 (FGF2), sonic hedgehog (SHH), bone morphogenetic protein (BMP) and reactive oxygen species (ROS) are pivotal regulators of fibroblast activation [6, 24–26]. For example, quiescent pancreatic stellate cells (PSCs) and hepatic stellate cells (HSCs), tissue-resident fibroblasts of the pancreas and liver, can acquire a myofibroblast-like phenotype including characteristics such as α-SMA expression when activated by TGF-β and PDGF and can then transform into CAFs [27, 28]. Mesenchymal stem cells (MSCs) might be another source of CAFs [29]. By fluorescent protein labeling, Raz et al. found that bone marrow-derived MSCs can be recruited and reprogrammed into different CAF subsets [30]. In breast cancer, osteopontin mediates the MZF1-TGF-β1-dependent differentiation of MSCs into CAFs [31]. *HOXA9* expressed in epithelial ovarian cancer cells induces adipose and bone marrow-derived MSCs to acquire the CAF phenotype through transcriptional activation of the gene encoding TGF-β2 [32]. These studies indicated that TGF-β plays an important role in this process. In prostate cancer, MSCs can differentiate into CAFs after activation by C-X-C motif chemokine receptor 6 (CXCR6) and its ligand CXCL16 [33]. Additionally, the recruitment and transformation of MSCs also depend on CXCL12, CCL2 and CCL5 secreted in the TME [29, 34]. CAFs might also be differentiated from nonfibroblast lines such as epithelial cells [35, 36], endothelial cells [37], adipocytes [38], pericytes [39] and smooth muscle cells [40]. Epithelial cells and endothelial cells transdifferentiate into CAFs through epithelial-to-mesenchymal transition (EMT) or endothelial-to-mesenchymal transition (EndoMT), after which they express an abundance of mesenchymal markers such as α-SMA and FAP [41, 42].

In summary, the CAF population is composed of multiple cell subsets and may have diverse phenotypes and functions. However, overall, the exact origins of CAFs have not been fully clarified. CAFs express a variety of proteins, such as α-SMA, PDGF receptor-α/β (PDGFRα/β), FAP, fibronectin, kindlin-2, tenascin C and integrin-α11 [43–46]. However, the expression levels of these proteins vary among different CAF subsets, and none of these proteins is specific to NFs or CAFs [47]. To further identify the origins of CAFs, researchers have used mouse models with genetic lineage tracing and fluorescence tagging, which can suitably describe the models of disease progression [30, 48]. Although these models have undergone technical progress, identifying the exact biological origin of CAFs remains difficult because of the lack of specific markers for NFs and CAFs [49, 50].

## Heterogeneity and plasticity of CAFs

As previously described, the CAF population is composed of multiple cell subsets, which typically exhibit heterogeneity. In pancreatic cancer, a study grouped CAFs into two subsets: myCAFs and iCAFs [9]. MyCAFs are located adjacent to cancer cells, are characterized by a high level of α-SMA expression and exhibit a matrix-producing contractile phenotype. In contrast, iCAFs are located further from cancer cells and are recognized by their high expression of tumor-promoting cytokines and chemokines such as interleukin-6 (IL-6). Further, Elyada et al. confirmed the existence of myCAFs and iCAFs in pancreatic cancer by single-cell RNA sequencing [51]. Interestingly, in that study, the researchers also identified a new subset of CAFs and defined these cells as “antigen-presenting CAFs” (apCAFs), which express MHC-II and CD74 but do not express classical costimulatory molecules. ApCAFs can activate CD4+ T cells in an antigen-specific manner, indicating their immunoregulatory ability. In another study, CAFs in pancreatic cancer were divided into at least four subtypes based on a transcriptomic analysis [52]. Subtype A cells labeled with periostin were found in the invasive front of the primary tumor, which is important for the formation of tumor capsule and metastasis. Subtype B cells labeled with myosin-11 were enriched in larger tumors and were associated with lymph node metastasis and poor prognosis. Subtype C cells labeled with podoplanin were enriched in a variety of immune-related pathways and seem to have an immunogenic profile and are related to a better prognosis. In addition, in breast cancer, a recent study identified four CAF subpopulations (S1-4) with different distributions and unique properties among breast cancer subtypes [53]. Among these subpopulations, S1-CAFs, which overexpress FAP, can attract T cells and promote their differentiation into Treg cells, thus contributing to an immunosuppressive TME. In contrast to promoting tumor growth, some subtypes of CAFs may be able to suppress tumor progression. For example, Rhim et al. found that the number of α-SMA + myofibroblasts was decreased in the TME of mice with Shh-deficient pancreatic ductal adenocarcinoma (PDAC), resulting in poor differentiation and stronger invasiveness [54]. Additionally, Ozdemir et al. demonstrated that depletion of α-SMA + fibroblasts leads to more invasive and undifferentiated tumors [50]. In some cases, different subtypes of CAFs in the same TME may have opposite effects on cancer progression. For instance, two CAF subtypes that differentially express CD146 play conflicting roles in the responsiveness of breast cancer to endocrine therapy [55]. Importantly, Givel et al. grouped CAFs into four subpopulations in mesenchymal high-grade serous ovarian cancer based on CD29, FAP, FSP1, and SMA protein levels [56]. CAF-S1 and CAF-S4 fibroblasts were defined as activated CAFs because of their high SMA expression levels, while CAF-S2 and CAF-S3 fibroblasts were considered to be nonactivated CAFs with low levels of SMA. CAF-S1 fibroblasts can improve the survival of CD25^+^FOXP3^+^ T lymphocytes and result in immunosuppression, which is mediated by the high expression of CXCL12β in CAF-S1. The different levels of CXCL12β in CAF-S1 and CAF-S4 fibroblasts lead to different functions among CAF subpopulations, which ultimately affects the prognosis of cancer patients [56, 57].

Due to the heterogeneous phenotypes, functions and spatial distribution of CAFs, researchers began to consider whether different subtypes can be interconverted—that is, whether CAFs exhibit plasticity. CAFs were found to be able to transition from the α-SMA+ and IL-6-producing state through TGF-β-dependent manipulation [58]. Additionally, prostate CAFs can acquire a tumor-like phenotype via a novel lipid-microtubule-organizing center (MTOC) axis regulated by pigment epithelium-derived factor (PEDF) [59]. In pancreatic cancer, the activation of the JAK/STAT signaling pathway induces the iCAFs phenotype, while JAK inhibitors can transform iCAFs into myCAFs; TGF-β signaling can also antagonize IL1-induced JAK/STAT signaling, thus inhibiting the iCAFs phenotype [60]. The phenotypic and functional transformation of CAFs reflects their instability and plasticity, which is a potential target for cancer therapy. However, considering the profound adaptability of fibroblasts to direct, local and long-term stimulatory factors, it is difficult to determine whether targeting specific CAF subsets will have lasting effects [61]. Many signaling pathways, such as EGFR signaling, Wnt signaling, Hippo signaling and TGF-β signaling, have been shown to play a key role in the regulation of CAF heterogeneity [57]. These signaling pathways may be another important direction for CAF-targeted therapy, and further studies of these pathways may help improve our understanding of CAF heterogeneity.

## MiRNAs in CAFs

### Biosynthesis and mechanisms of miRNAs

MiRNAs are a class of sncRNAs encoded by endogenous genes with a length of approximately 22 nt. The human genome contains more than 2000 miRNAs, approximately 70% of which are related to protein-coding genes and are cotranscribed [62]. The miRNA biosynthesis pathway is complex. The miRNA host gene is transcribed to produce a primary RNA (pri­miRNA), which has a hairpin structure and a poly(A) tail, by either RNA polymerase II or RNA polymerase III [63, 64]. In the nucleus, the pri-miRNA is processed first by a protein complex consisting of two proteins, Drosha and DiGeorge critical region 8 (DGCR8, also known as Pasha), which are an RNase III protein and a double-stranded RNA-binding protein, respectively. DGCR8 positions the Drosha cutting site 11 base pairs (bp) from the base of the hairpin stem, and the pri-miRNA is then processed into a 60–70 nt precursor miRNA (pre-miRNA) by Drosha [65, 66]. Later, the pre-miRNA is transported from the nucleus to the cytoplasm with the assistance of Exportin-5 in complex with Ran-GTP [67, 68]. In the cytoplasm, the pre-miRNA is recognized by Dicer, a member of the RNase III enzyme family, and is processed into a 20 nt double-stranded miRNA via cutting and modification of the stem-loop structure [69]. The resulting double-stranded miRNA binds to Argonaute proteins to form the RNA-induced silencing complex (RISC), in which one of the miRNA strands binds to the 3′ UTR of the target mRNA through the incorporation of protein complexes, while the other miRNA strand is degraded or released [13, 70].

The regulatory effect of miRNAs on gene expression occurs at the posttranscriptional level through two main mechanisms, which depend on the complementarity between the miRNA and target gene mRNA sequences. If the miRNA sequence is nearly perfectly complementary to that of the target mRNA—for example, in plants—the miRNA forms a completely complementary double-stranded RNA with the sequence in the ORF of the mRNA. Then, the RISC degrades this double-stranded mRNA and silences gene expression posttranscriptionally. Otherwise, in most cases, the miRNA forms an incompletely complementary hybrid double-stranded RNA with the 3′ UTR sequence of the target mRNA, and the RISC binds tightly to this hybrid RNA to specifically inhibit gene expression [13, 71, 72]. Importantly, one miRNA can regulate multiple mRNAs and, conversely, one mRNA can be targeted by several miRNAs [62]. In addition, as the understanding of miRNAs has deepened, many unconventional functions of miRNAs have been gradually discovered, such as binding to the 5′ UTR of mRNAs or binding directly to Toll-like receptors [73–75].

### MiRNAs in the formation and activation of CAFs

Tumor progression requires stromal support to maintain tumor cell proliferation, migration and metastasis [4]. For tumor cells, it is necessary to reprogram inactivated fibroblasts into tumor-promoting CAFs to maintain tumor progression. The activation and maintenance of CAFs may be related to epigenetic regulation [76], gene mutation [77, 78] and cytokine stimulation in the TME [79]. MiRNA dysregulation is vital to these processes and has been observed in CAFs in various cancers, including breast cancer [80], ovarian cancer [81] and lung cancer [82]. Thus, miRNAs are considered to be pivotal regulatory factors for the formation and activation of CAFs, and many studies have confirmed this belief (Table 1).

**Table 1:** MicroRNAs involved in the formation and activation of cancer-associated fibroblasts

Cancer type	miRNA	Expression	Upstream signaling	Target molecules or pathways	References
*miRNA dysregulation*					
Breast cancer	miR-200b/c miR-221b	↓ ↑	TGF-β	DNMT3B, miR-200s	[85]
Breast cancer	miR-21	↑	CCL18/NF-κB	PTEN/AKT axis	[90]
Breast cancer	miR-222	↑	–	LBR	[202]
Breast cancer	miR-200b/c	↓	–	IKKβ/NF-κB axis	[203]
Breast cancer	miR-200s	↓	TGF-β1	Fli-1, TCF12	[138]
Colorectal cancer	miR-21	↑	TGF-β	–	[83]
Gastric cancer	miR-149	↓	–	IL-6	[87]
Lung cancer	miR-1, miR-206 miR-31	↓ ↑	–	FOXO3a/VEGFA/CCL2 axis	[89]
Pancreatic cancer	miR-21	↑	–	TGF-β	[84]
Prostate cancer	miR-205	↓	–	IL-6	[88]
-	miR-21	↑	–	Smad7/ TGF-β1	[204]
*Exosomal miRNA*					
Breast cancer	miR-125b	↑	–	TP53INP1/TP53	[95]
Breast cancer	miR-146a	↑	–	TXNIP/Wnt axis	[205]
Breast cancer	miR-9	↑	–	–	[206]
Hepatocellular carcinoma	miR-21	↑	–	PTEN/PDK1/AKT axis	[96]
Hepatocellular Carcinoma	miR-1247-3p	↑	–	B4GALT3, β1-integrin/NF-κB axis	[97]
Melanoma	miR-211	↑	–	IGF2R, MAPK	[98]
Pancreatic cancer	miR-155	↑	–	TP53INP1	[207]

Induction by growth factors, cytokines and chemokines is an important pathway of CAF formation and activation. It was found that colorectal cancer-derived TGF-β increases the expression of miR-21, which upregulates α-SMA and promotes the activation of CAFs [83]. A recent study in an orthotopic transplantation model proved again that high expression of miR-21 in the stroma can allow the conversion of NFs into SMA + CAFs and affect tumor growth by activating the TGF-β signaling pathway [84]. In NFs treated with exogenous TGF-β, the decrease in miR-200b/c expression and the increase in miR-221b expression induce DNMT3B expression at a stable and high level. Promoter methylation of miR-200s is induced by DNMT3B, leading to a decrease in miR-200s expression, which results in the activation of CAFs [85]. IL-6 is a chemokine present in the TME that may also be crucial for CAF activation [86]. Li et al. reported that in gastric cancer, miR-149 inhibits the activation of fibroblasts by targeting IL-6 mRNA and reducing its expression [87]. In prostate cancer, miR-205 inhibits tumor-driven fibroblast activation by reducing the secretion of proinflammatory cytokines such as IL-6 [88]. In addition, Shen et al. revealed that CCL-2 is also a target of miRNA and is crucial for CAF activation. Downregulation of miR-1 and miR-206 and upregulation of miR-31 reprogram NFs into CAFs by mediating FOXO3a/VEGFA/CCL2 signaling [89]. Regarding breast cancer, tumor-associated macrophages induce myofibroblast differentiation and increase the expression of α-SMA through a CCL18/NF-κB/miR-21/PTEN/AKT signaling axis [90].

In addition, miRNAs contained in exosomes are pivotal for the formation and activation of CAFs. Exosomes are membrane-coated vesicles derived from the endosomal system [91, 92] that are 30–100 nm in diameter and contain nucleic acids, proteins and lipids to mediate communication among cancer cells, CAFs and other cells in the TME [93, 94]. Recently, Vu et al. confirmed by RNA sequencing that miR-125b is secreted by the mouse triple negative breast cancer cell lines 4T1 and 4TO7, and *TP53INP1* suppression induced by exosomal miR-125b is one cause of fibroblast activation in a mouse tumor model [95]. Further, exosomal miR-125b also activates human fibroblasts by suppressing the expression of *TP53INP1* and *TP53*. In hepatocellular carcinoma (HCC), exosomal miR-21 secreted from HCC cells targets *PTEN* directly, leading to the activation of PDK1/AKT signaling in HSCs and the transformation of these cells into CAFs [96]. Moreover, exosomal miR-1247-3p secreted from highly metastatic HCC cells targets *B4GALT3* and activates the β1-integrin/NF-κB axis in fibroblasts, thus resulting in the transformation of NFs into CAFs [97]. In melanoma, a study demonstrated that the MAPK signaling pathway is activated under the effect of melanosome-derived miR-211 via inhibition of the tumor suppressor *IGF2R*, resulting in the acquisition of a CAF phenotype by NFs [98].

### MiRNAs in the metabolic reprogramming of CAFs

CAFs have been found to secrete a large number of metabolites, including L-lactate, ketone bodies, fatty acids, amino acids and tricarboxylic acid (TCA) cycle intermediates, which can be taken up by cancer cells and support their growth [11, 99–101]. Many studies have reported that cancer cells modulate the metabolic reprogramming of CAFs through ROS and miRNAs; this process is described as the reverse Warburg effect and plays an important role in cancer progression [102–104].

MiR-210 is considered to be a hypoxia-related miRNA. In fibroblasts cultured under hypoxia, the expression of miR-210 was found to be increased sixfold. Overexpressed miR-210 can participate in the metabolic reprogramming of CAFs, thus promoting the production of L-lactate, ketone bodies and other metabolites to promote the growth of cancer cells [105]. Zhang et al. revealed that miR-424 is related to TGF-β-induced downregulation of IDH3α (a key rate-limiting enzyme in the TCA cycle) in CAFs. Downregulation of IDH3α leads to metabolic reprogramming from oxidative phosphorylation to glycolysis in CAFs and increases the expression of glycolysis-related enzymes such as GLUT1, HK2 and PFKM. Moreover, in CAFs overexpressing miR-424, lactate production and glucose uptake can be increased and oxygen consumption can be suppressed via an increase in HIF1α stability [106]. Regarding exosome-derived miRNAs, Yan et al. showed that the metabolic characteristics of CAFs are changed by exosomal miR-105 according to different metabolic environments through activation of the myc signaling pathway. After miR-105-mediated reprogramming, CAFs exhibit enhanced glucose and glutamine metabolism to fuel neighboring cancer cells when nutrients are adequate. Under nutrient-deficient conditions and accumulation of metabolic byproducts, CAFs can convert metabolic waste products into energy-rich metabolites [103]. This study further demonstrates the importance of miRNAs in regulating the metabolism of CAFs.

### MiRNAs in the effect of CAFs on cancer cells

**Fig. 2:**
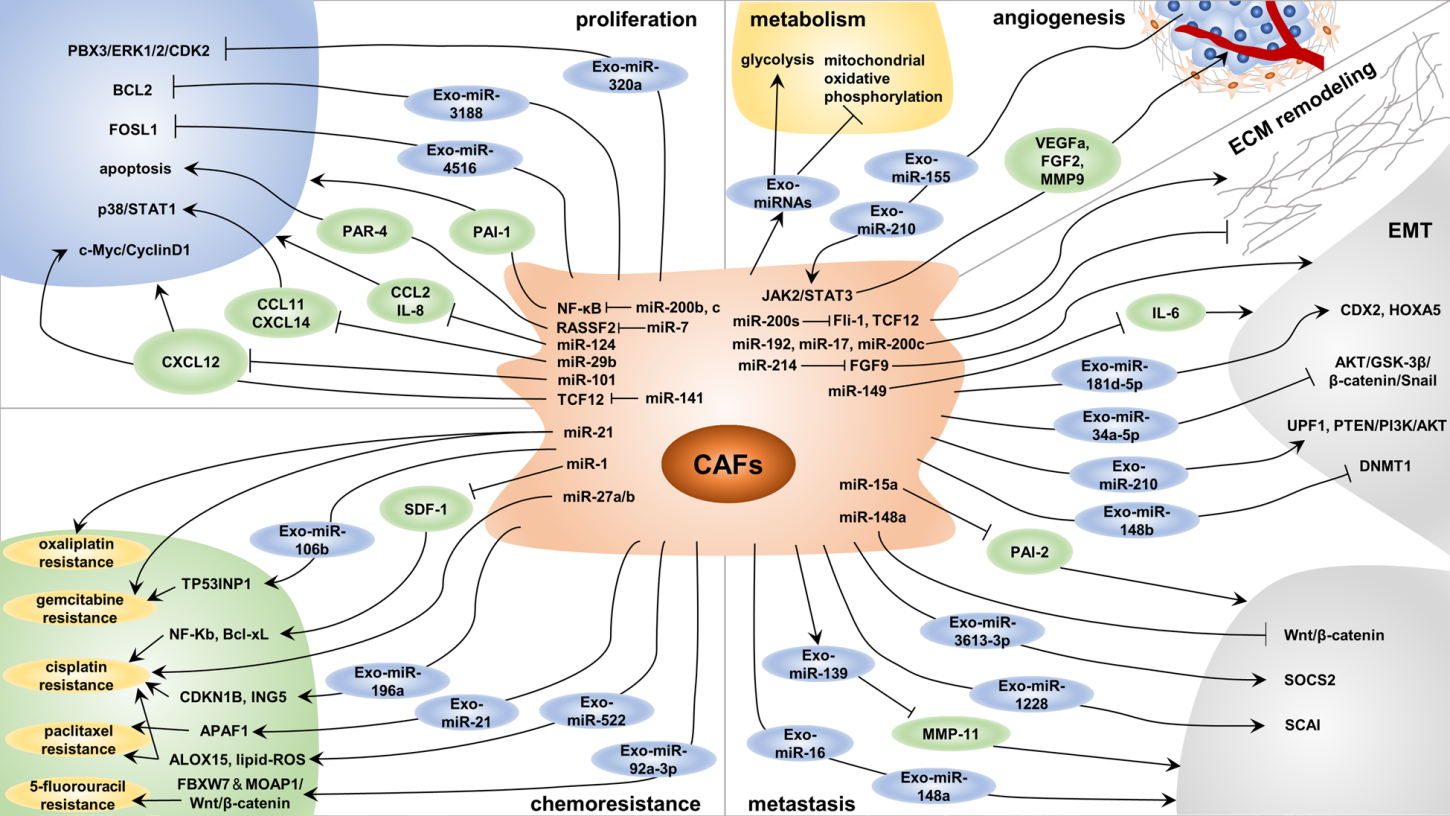
MicroRNAs (miRNAs) in the effect of cancer-associated fibroblasts (CAFs) on cancer cells. MiRNA dysregulation in CAFs and exosomal miRNAs derived from CAFs affect tumor cell proliferation, metabolism, angiogenesis, metastasis and chemoresistance via many mechanisms and ultimately modulate tumor progression

The crosstalk between cancer cells and the TME is considered to be essential in the progression of cancer [107]. The interaction of CAFs and cancer cells is mediated through a complex signaling network, and each cell type affects the function of the other via synergistic or antagonistic signaling axes [8]. Numerous in vitro and in vivo experiments have revealed that CAFs can influence the proliferation, stemness, metabolism, angiogenesis, metastasis and therapeutic resistance of cancer by secreting cytokines, growth factors and exosomes [6, 108]. Furthermore, accumulating evidence has shown that miRNAs are crucial for the effects of CAFs on cancer cells (Fig. 2 and Table 2). In this section, these studies are reviewed.

**Table 2:** MicroRNAs in the effect of cancer-associated fibroblasts on cancer cells

Cancer type	miRNA	Expression	Function	Upstream signaling	Target molecules or pathways	References
*miRNA dysregulation*						
Breast cancer	miR-141	↓	Proliferation	TGF-β1/DNMT3B	TCF12/ CXCL12/ c-Myc/CyclinD1	[109]
Breast cancer	miR-29b	↓	Proliferation, Chemoresistance	–	CCL11, CXCL14, p38/STAT1	[111]
Breast cancer	miR-200b, c	↓	Proliferation, EMT, Invasion	–	IKKβ/NF-κB/PAI-1	[113]
Breast cancer	miR-200s	↓	Metastasis	–	Fli-1, TCF12	[138]
Cholangiocarcinoma	miR-15a	↓	Migration	–	PAI-2	[140]
Colorectal cancer	miR-192, miR-17, miR-200c	↓	Invasion	–	ECM Associated Genes	[139]
Colorectal cancer	miR-21	↑	Proliferation, Invasion, Chemoresistance	–	RECK, MMP2	[83]
Endometrial cancer	miR-148a	↓	Proliferation, Migration	–	WNT10B, Wnt/β-catenin	[141]
Esophageal cancer	miR-27a/b	↑	Chemoresistance	–	TGF-β	[156]
Gastric cancer	miR-214	↓	EMT, Migration	–	FGF9	[143]
Gastric cancer	miR-149	↓	Proliferation, EMT	COX-2/PGE2, DNA Hypermethylation	IL-6	[87]
Head and Neck cancer	miR-7	↑	Proliferation, Migration	-	RASSF2, PAR-4	[114]
Lung cancer	miR-101	↓	Proliferation	-	CXCL12	[82]
Lung cancer	miR-1	↓	Proliferation, Chemoresistance	-	SDF-1, CXCR4, NF-κB, Bcl-xL	[155]
Oral carcinoma	miR-124	↓	Proliferation, Migration	DNA Hypermethylation	CCL2, IL-8	[110]
Pancreatic cancer	miR-21	↑	Chemoresistance	–	Desmoplasia	[154]
Prostate cancer	miR-15, miR-16	↓	Proliferation, Migration	–	FGF-2, FGFR1	[112]
Prostate cancer	miR-210	↑	Angiogenesis	Hypoxia	HUVECs	[105]
*Exosomal miRNA*						
Breast cancer	miR-4516	↓	Proliferation	–	FOSL1	[115]
Breast cancer	miR-205	↓	Angiogenesis	–	YAP1/ IL-11, IL-15/ STAT3	[135]
Breast cancer	miR-181d-5p	↑	Proliferation, EMT	–	CDX2/HOXA5	[144]
Breast cancer	miR-16, miR-148a	↑	Metastasis	FAK	–	[148]
Breast cancer	miR-3613-3p	↑	Proliferation, Metastasis	–	SOCS2	[149]
Colorectal cancer	miR-21	↑	Metastasis	–	–	[151]
Colorectal cancer	miR-92a-3p	↑	EMT, Metastasis, Chemoresistance	–	FBXW7 and MOAP1/Wnt/β-catenin	[122]
Endometrial cancer	miR-148b	↓	EMT, Metastasis	–	DNMT1	[145]
Gastric cancer	miR-139	↓	Proliferation, Metastasis	–	MMP11	[118]
Gastric cancer	miR-522	↑	Metabolic Reprogramming, Chemoresistance	USP7/hnRNPA1	ALOX15, lipid-ROS	[127]
Head and neck cancer	miR-3188	↓	Proliferation	–	BCL2	[116]
Head and neck cancer	miR-196a	↑	Proliferation, Chemoresistance	hnRNPA1	CDKN1B, ING5	[157]
Hepatocellular carcinoma	miR-320a	↓	Proliferation, EMT, Metastasis	–	PBX3/ERK1/2/CDK2	[117]
Lung cancer	miR-210	↑	Angiogenesis	–	JAK2/STAT3	[134]
Lung cancer	miR-210	↑	EMT, Migration	–	UPF1, PTEN/PI3K/AKT	[146]
Melanoma	miR-155	↑	Angiogenesis	–	SOCS1/JAK2/STAT3	[133]
Oral cancer	miR-34a-5p	↓	Proliferation, EMT, Metastasis	–	AXL, AKT/GSK-3β/β-catenin/Snail	[147]
Osteosarcoma	miR-1228	↑	Migration	–	SCAI	[150]
Ovarian cancer	miR-21	↑	Invasion, Chemoresistance	–	APAF1	[158]
Ovarian cancer	miR-98-5p	↑	Chemoresistance	–	CDKN1A	[159]
Pancreatic cancer	miR-146a	↑	Proliferation, Chemoresistance	Gemcitabine	–	[119]
Pancreatic cancer	miR-106b	↑	Chemoresistance	–	TP53INP1	[160]

#### Proliferation

The proliferation of cancer cells is the most important and basic step in tumor progression. As mentioned earlier, CAFs can regulate the proliferation of cancer cells by secreting various cytokines or chemokines under the regulation of miRNAs. For example, signaling through the TGF-β1/DNMT3B/miR-141 axis can increase the expression of TCF12, which promotes CXCL12 secretion by CAFs, thus activating the c-myc/CyclinD1 signaling pathway in cancer cells and finally enhancing their proliferation [109]. In addition, Zhang et al. found that miR-101 in CAFs inhibits tumor growth and metastasis by targeting CXCL12 [82]. In oral carcinoma, downregulation of miR-124 in CAFs facilitates the proliferation of cancer cells by targeting CCL2 and IL-8 [110]. Importantly, the increases in the proliferation and drug resistance of breast cancer cells are related to downregulation of miR-29b in CAFs, which leads to increased secretion of CCL11 and CXCL14 possibly partially via activation of the p38-STAT1 pathway in cancer cells [111].

FGF-2 and its receptor FGFR1 can enhance tumor cell proliferation and migration by acting on stromal cells and cancer cells. Musumeci et al. revealed that downregulation of miR-15 and miR-16 in CAFs promotes tumor growth by reducing the posttranscriptional inhibition of *FGF-2* and *FGFR1* [112]. The expression of plasminogen activator inhibitor 1 (PAI-1), an important intermediary in the crosstalk between CAFs and epithelial tumor cells, in CAFs is closely related to the miR-200b, c/NF-κB axis and affects the proliferation of cancer cells [113]. In addition, miRNAs can regulate the ability of CAFs to promote cancer cell apoptosis, thus affecting tumor growth. In head and neck cancers, overexpression of miR-7 in CAFs leads to downregulation of *RASSF2*, which greatly reduces the secretion of protease-activated receptor 4 (PAR-4), a proapoptotic factor in cancer cells, thus leading to enhancement of cancer cell proliferation [114].

The role of CAF-derived exosomal miRNAs in tumor proliferation has also received increasing attention. Kim et al. found that a decrease in the miR-4516 level in CAF-derived exosomes enhances the proliferative ability of triple negative breast cancer cells by reducing the inhibition of *FOSL1*, a proliferation-related gene [115]. In head and neck cancers, loss of exosomal miR-3188 from CAFs promotes tumor growth by diminishing the inhibition of B-cell lymphoma 2 (BCL2) [116]. The miR-320a/PBX3 axis downregulates the expression of cyclin-dependent kinase 2 (CDK2) by targeting ERK1/2 to reduce MAPK signaling pathway activation, thus inhibiting the proliferation of HCC cells [117]. However, the amount of exosomal miR-320a derived from CAFs was found to be significantly decreased, which facilitated the growth and progression of HCC. Xu et al. revealed that the growth and metastasis of gastric cancer cells are inhibited by exosomal miR-139 derived from CAFs, an effect mediated by downregulation of matrix metalloproteinase (MMP) 11 [118]. Interestingly, the level of CAF-derived exosomal miR-146a is increased by treatment with gemcitabine, while blocking exosome secretion can inhibit the proliferation and survival of PDAC cells [119].

#### Stemness

A population of cells in tumors play an irreplaceable role in tumor initiation, progression and therapeutic resistance, and have high potential for self-renewal and differentiation. These cells are defined as cancer stem cells (CSCs), and the acquisition and maintenance of their stemness are affected by many factors. The ability of CAFs to promote stemness has been widely studied in several tumor types [12, 120], but there are relatively few studies on the role of CAF-derived miRNAs in stemness regulation. In breast cancer, Donnarumma et al. identified three significantly upregulated miRNAs (miR-21-5p, miR-378e, and miR-143-3p) in CAF-derived exosomes by miRNA-seq [121]. CAF-derived exosomal miR-21-5p, miR-378e and miR-143-3p promote the stemness properties and EMT phenotype of tumor cells. Among them, miR-21-5p mainly plays a role in EMT induction, while miR-378e and miR-143-3p have a stronger effect on promoting stemness; however, the specific mechanisms of these processes are not clear. Another study found that exosomal miR-92a-3p enhances tumor stemness by targeting *FBXW7* and *MOAP1* [122]. Additionally, by promoting the ubiquitination degradation of β-catenin and mitochondrial apoptosis, FBXW7 and MOAP1 can reverse the oncogenic effect of miR-92a-3p. In addition to the direct effect of CAF-derived miRNAs on stemness, CAFs can also indirectly promote stemness by regulating the expression of specific miRNAs in tumor cells [123]. Overall, miRNAs represent an important way for CAFs to regulate stemness and are worthy of more in-depth and extensive exploration.

#### Metabolism

Reprogramming of energy metabolism is a hallmark of cancer cells [124]. A well-characterized dysregulation of cancer metabolism is the Warburg effect, or aerobic glycolysis, which refers to the preference for oxygen-independent glycolysis and lactate secretion even in the presence of sufficient oxygen [125]. Although mitochondrial oxidative phosphorylation can produce more energy than glycolysis, cancer cells are thought to transfer glycolytic intermediates into biosynthetic pathways to produce amino acids, nucleotides and lipids that support cell growth via aerobic glycolysis [126]. As mentioned earlier, metabolic reprogramming CAFs can provide these metabolites for cancer cell growth. Moreover, CAF-derived miRNAs play an important role in regulating cancer cell metabolism. Exosomes derived from CAFs can inhibit mitochondrial oxidative phosphorylation in cancer cells through miRNAs and the transfer of substrates, and glycolysis and glutamine-dependent reductive carboxylation are then upregulated in cancer cells [101]. A recent study demonstrated that exosomal miR-522 derived from CAFs is a potential inhibitor of arachidonate lipoxygenase 15 (ALOX15), which is closely related to the production of lipid-ROS in gastric cancer [127]. These lines of evidence indicate that miRNA-regulated metabolic crosstalk between CAFs and cancer cells affects the progression of cancer via multifarious mechanisms. Improving our knowledge of these processes may open new avenues for the treatment of cancer.

#### Angiogenesis

Tumor angiogenesis plays a critical role in tumor progression and metastasis [128]. CAFs have been found to facilitate tumor angiogenesis by secreting a variety of angiogenic factors, such as vascular endothelial growth factor (VEGF) and FGF2 [129, 130]. Interestingly, some studies have suggested that miRNAs, which can regulate the secretion of VEGF and even control the function of endothelial cells, may also be important regulators of tumor angiogenesis [131, 132]. Thus, some researchers have begun to explore whether miRNAs contribute to CAF-mediated angiogenesis. High vascularization is one of the main features of melanoma. Exosomal miR-155 derived from melanoma cells inhibits the expression of suppressor of cytokine signaling 1 (SOCS1) by targeting its 3′ UTR and activates the JAK2/STAT3 signaling pathway. This pathway upregulates the expression of VEGFa, FGF2 and MMP9 in fibroblasts and then influences the angiogenic effect of CAFs [133]. A recent study has shown that exosomal miR-210 derived from lung cancer cells acts as a proangiogenic factor in CAFs also by modulating the JAK2/STAT3 pathway [134]. In addition, hypoxia-induced overexpression of miR-210 can induce the conversion of NFs into CAFs, further affecting the recruitment of endothelial progenitor cells and the capillary formation by human umbilical vein endothelial cells (HUVECs) [105]. In breast cancer, a decreased level of exosomal miR-205 induces activation of the target gene *YAP1* in CAFs, which promotes HUVEC tube formation and sprouting [135]. The expression of IL-11 and IL-15 is specifically enhanced by YAP1, while their release by CAFs activates STAT3 signaling in HUVECs, thus promoting angiogenesis. The current studies indicate that miRNAs from CAFs affect tumor angiogenesis mainly through STAT3 signaling, and further exploration is required to determine whether other pathways also contribute to this process.

#### Invasion, migration and metastasis

CAFs promote tumor invasion, migration and metastasis in many ways, and the regulation of ECM remodeling is one of the important mechanisms [136]. A recent study showed that a matrix-dependent mechano-sensitization of EGF signaling in cancer cells can drive the collective invasion of squamous cell carcinoma (SCC) cells [137]. SCC cells activate EGFR under the condition of tumor stiffness and lead to Ca^2+^-dependent regulation of Cdc42 small GTPase activity, which results in MLC2 phosphorylation. Then the MLC kinase regulates actomyosin-dependent ECM remodeling induced by CAFs and ultimately promotes the invasion of SCC [57, 136, 137]. MiRNA dysregulation in CAFs can also mediate tumor cell invasion and metastasis by regulating ECM remodeling. Downregulation of miR-200s in CAFs increases the expression of Fli-1 and TCF12, which facilitates ECM remodeling and the production of parallel pattern-oriented ECM fibers by CAFs, thus accelerating the invasion and metastasis of tumor cells [138]. Mechanistically, inhibition of Fli-1 or TCF12 in CAFs counteracts the contractile activity induced by CAFs, but overexpression of Fli-1 or TCF12 in NFs increases the contractile activity of these NFs. In another study, a combined regression model was used to identify the subgroup-specific miRNAs and functional gene sets in colorectal cancer, and that the results indicated that some miRNAs can regulate ECM target genes [139]. Further studies showed that the high expression of miR-192, miR-17 and miR-200c in fibroblasts can regulate ECM target gene expression at the protein level, thus regulating ECM remodeling. Finally, a coculture experiment confirmed that the high expression of miR-192, miR-17 and miR-200c in colon CAFs decreases the invasive ability of colorectal cancer cells [139]. In addition, PAIs are important molecules in tumor invasion and metastasis. The expression of PAI-1 in CAFs is increased by the miR-200b, c/NF-κB axis, which induces polarity changes, such as a decrease in E-cadherin and an increase in Vimentin, in cancer cells, thus enhancing their invasive ability [113]. Similarly, through coculture experiments and wound healing assays, it has been proven that the miR-15a/PAI-2 axis promotes the migration of cholangiocarcinoma cells [140]. Furthermore, silencing of miR-148a in CAFs promotes the migration of endometrial cancer cells by targeting *Wnt10B* to activate the Wnt/β-catenin pathway [141]. As shown in Table 2, other miRNAs in CAFs are also involved in tumor invasion, migration and metastasis in vitro or in vivo, as confirmed by coculture or xenograft experiments.

EMT, which can enhance the initiating and metastatic potential of cancer cells, is an important process affecting tumor metastasis [142]. Downregulation of miR-214 in gastric CAFs can promote EMT of gastric cancer cells, which is characterized by increased expression of E-cadherin and decreased expression of N-cadherin, Vimentin and Snail. This promotive effect may be achieved through the direct targeting of *FGF9* by miR-214 and eventually lead to the migration and invasion of gastric cancer cells [143]. Importantly, CAFs can also enhance the EMT and stem-like characteristics of gastric cancer cells through the miR-149/IL-6 axis [87]. Gastric cancer cells induce the activation of the COX2/PGE2 signaling pathway and enhance the production of PGE2, which leads to epigenetic silencing of miR-149 in CAFs, thus ultimately facilitating tumor progression. CAF-derived exosomal miRNAs are also important for EMT in cancer cells. A study in a xenograft model proved that exosomal miR-181d-5p derived from CAFs induces EMT in breast cancer by regulating *CDX2* and *HOXA5* in vivo [144]. In endometrial cancer, exosomal miR-148b derived from CAFs inhibits EMT and invasion of cancer cells by directly targeting *DNMT1*, thus regulating cancer metastasis in vitro and in vivo [145]. In lung cancer, CAF-derived exosomal miR-210 can promote EMT by targeting *UPF1* to activate the PTEN/PI3K/AKT signaling pathway [146]. Additionally, loss of exosomal miR-34a-5p activates the AKT/GSK-3β/β-catenin/Snail signaling pathway by directly targeting *AXL*, ultimately accelerating EMT and invasion of cancer cells [147].

CAF-derived exosomal miRNAs can also directly regulate tumor invasion and metastasis by targeting related genes. A recent study demonstrated that the regulation of miR-16 and miR-148a in CAF-derived exosomes by FAK signaling contributes to alterations in the ability of CAFs to affect the activity and metastasis of breast cancer cells [148]. Additionally, CAF-derived exosomes with decreased levels of miR-3613-3p lead to suppression of proliferation and metastasis via targeting of *SOCS2* [149]. In gastric cancer, an increase in MMP11 in CAF exosomes induces the migration and metastasis of cancer cells, while CAF-derived exosomal miR-139 negatively regulates the level of MMP11 [118]. As summarized in Table 2, other exosomal miRNAs, such as miR-1228 in osteosarcoma [150] and miR-21 in colorectal cancer [151], can also participate in the regulation of tumor metastasis by targeting different genes.

#### Therapeutic resistance

Despite recent progress in chemotherapy, molecular therapy and immunotherapy, many patients do not benefit from these treatment modalities [152]. Accumulating studies have proven that CAFs are closely related to therapeutic resistance and poor prognosis [8]. Therapeutic resistance of tumors can be induced by CAFs via many mechanisms, including the secretion of paracrine signaling mediators, such as cytokines and exosomes; prevention of drug delivery; reprogramming of metabolism; and regulation of signaling pathways [7, 34, 122, 153]. MiRNAs also play an indispensable role in these processes.

The effect of miRNA dysregulation in CAFs on tumor drug resistance has been widely studied. As early as 2013, Bullock et al. found that coculture with fibroblasts overexpressing miR-21 can protect colorectal cancer cells from apoptosis induced by oxaliplatin and increase the proliferative ability of these cells [83]. In addition, Zhang et al. proved through animal experiments that upregulation of miR-21 in CAFs can promote desmoplasia and increase gemcitabine resistance in PDAC [154]. In lung cancer, the expression of stromal cell-derived factor 1 (SDF-1) in CAFs is negatively regulated by miR-1. SDF-1 promotes the proliferation and cisplatin resistance of lung cancer cells through CXCR4-modulated NF-κB and Bcl-xL signaling pathways [155]. In a study on esophageal cancer, coculture with miR-27a/b-transfected NFs was found to reduce the sensitivity of cancer cells to cisplatin [156].

The role of exosomal miRNAs in CAF-mediated therapeutic resistance has been continuously reported in recent years. In gastric cancer, cisplatin and paclitaxel accelerate the secretion of exosomal miR-522 from CAFs by activating the USP7/hnRNPA1 axis, which results in inhibition of ALOX15. Downregulation of ALOX15 reduces the accumulation of lipid-ROS in cancer cells, which ultimately leads to acquired drug resistance [127]. In colorectal cancer, exosomal miR-92a-3p secreted from CAFs activates the Wnt/β-catenin signaling pathway and inhibits mitochondrial apoptosis through direct inhibition of *FBXW7* and *MOAP1*, resulting in 5-fluorouracil/oxaliplatin resistance [122]. Qin et al. reported that exosomal miR-196a enhances the proliferation and cisplatin resistance of head and neck cancer cells by promoting G1/S cell cycle transition and inhibiting apoptosis via targeting *CDKN1B* and *ING5* [157]. In addition, this study reconfirmed the importance of hnRNPA1 in mediating the formation of CAF-derived exosomes. As shown in Table 2, CAF-derived miR-21 and miR-98-5p in ovarian cancer and miR-146a and miR-106b in pancreatic cancer target different molecules but consistently mediate the process of tumor drug resistance [119, 158–160].

### MiRNAs in CAFs and cancer immunosuppression

The emergence of immunotherapy represents a major breakthrough in cancer treatment, but most patients show resistance to these therapies. Growing evidence has shown that the TME is one of the key determinants of the tumor immune response, and CAFs are closely associated with the efficacy of immunotherapy [161]. CAFs directly interact with immune cells by secreting growth factors, cytokines and chemokines, thus mediating and regulating the infiltration of immune cells [162]. In addition, CAFs can also create a physical immune barrier through ECM remodeling to exert immunomodulatory effects [163]. Although considerable evidence confirms that CAFs mainly play an immunosuppressive role, recent studies have demonstrated that some subpopulations of CAFs may also be related to the activation of the tumor immune response and to a better prognosis [52, 164]. Studies on the role of miRNAs in CAFs in cancer immunomodulation are also ongoing. A recent study found that in lung cancer tissues, the high expression of lncRNA PCAT-1 is related to the induction and infiltration of CAFs, which may be achieved via the immunosuppressive miR-182/miR-217 signaling [165]. Concurrently, the high expression of PCAT-1 is closely associated with the immunosuppression of lung cancer. Although further verification is still needed, this study suggests that CAFs may mediate lung cancer immunosuppression through ncRNAs. As mentioned earlier, Givel et al. grouped CAFs into four subpopulations [56]. Under the action of miR-141 and miR-200a, members of the miR-200 family, the expression of CXCL12β was downregulated in CAF-S4 fibroblasts and upregulated in CAF-S1 fibroblasts. CAFs with high expression of CXCL12β improve the survival in CD25^+^FOXP3^+^ T lymphocytes and result in immunosuppression. Additionally, CAF-S1 fibroblasts can also increase the content of CD25^+^Foxp3^+^ T lymphocytes, which is mediated by the high expression of B7-H3, CD73 and IL6 [56].

### MiRNAs in CAFs as biomarkers for cancer prognosis

The prognosis of cancer patients has always been the focus of attention. The discovery of effective prognostic biomarkers will undoubtedly contribute greatly to the treatment and management of cancer patients. Therefore, in addition to investigating the role of miRNAs in the interaction between CAFs and tumor cells, many studies have begun to consider the potential of miRNAs in CAFs to be prognostic biomarkers (Table 3). Interestingly, Lee et al. found that in colorectal cancer patients with distant metastasis, low expression of miR-21 in stromal fibroblasts in the periphery of the primary tumor was significantly associated with poorer overall survival (OS) [166], a conclusion quite different from those of Kunita et al. [167] and Kadera et al. [168]. To some extent, this discrepancy indicates that the prognostic value of miRNAs in CAFs needs further verification, and this need has launched a new direction for the further study of miR-21. In addition, as shown in Table 3, in some studies of CAF exosomes, researchers have reported the prognostic value of serum or plasma exosomal miRNAs [96, 157]. However, more studies are needed to prove whether the effects of these miRNAs are consistent with those of miRNAs in CAF-derived exosomes.

**Table 3:** MicroRNAs in cancer-associated fibroblasts as biomarkers for cancer prognosis

Cancer type	No. of samples	ncRNA	Dysregulation or exomosal	Method	Expression*	Result in	References
Colorectal cancer	170	miR-21	Dysregulation	ISH	↓	Poorer OS	[166]
Gastric cancer	120	miR-106b	Dysregulation	ISH	↑	Poorer OS	[208]
Gastric cancer	68	miR-143	Dysregulation	ISH and qRT-PCR	↑	Higher CSM	[209]
Gastric cancer	71	miR-145	Dysregulation	qRT-PCR	↑	Higher CSM	[210]
Head and neck cancer	74	miR-196a	Exomosal	qRT-PCR	↑	Poorer OS	[157]
Hepatocellular carcinoma	85	miR-21	Exomosal	qRT-PCR	↑	Poorer OS	[96]
Lung cancer	89	miR-21	Dysregulation	ISH	↑	Poorer OS	[167]
Lung cancer	134	miR-200a	Dysregulation	ISH	↓	Poorer OS	[211]
Pancreatic cancer	153	miR-21	Dysregulation	ISH	↑	Poorer OS	[168]
Oral squamous cell carcinoma	140	lncRNA FLJ22447	Dysregulation	qRT-PCR	↑	Poorer OS	[188]
Ovarian cancer	62	10 lncRNAs	Dysregulation	–	–	Poorer OS	[199]

OS, overall survival; CSM, cancer-specific mortality

*Refers to the expression level of the poor prognosis group in the case–control study

### Potential therapeutic approaches for targeting miRNAs and exosomes

#### Targeting miRNAs

Because miRNAs can play either a promotive or suppressive role in tumors, there are two main therapeutic approaches targeting miRNAs: reducing the expression of miRNAs that act as oncogenes (onco-miRNAs) and restoring the expression of miRNAs that act as tumor suppressors. The expression or function of onco-miRNAs can be inhibited by using single-stranded antisense RNAs, small molecule antagonists or miRNA sponges, while the expression or function of tumor suppressor miRNAs can be enhanced via double-stranded miRNA mimics [22]. To date, relatively few clinical trials of miRNA-targeted therapy in tumors have been conducted, and these trials have focused primarily on the following targets: miR-34a (NCT01829971, NCT02862145), miR-155 (NCT02580552, NCT03713320, NCT03837457) and miR-16 (NCT02369198). Although some trials have failed and most studies are still in the early stage, these studies still show the prospects of miRNA-targeted therapy, as well as some experiences worthy of consideration. In studies of miRNAs in CAFs, some scholars have also tried to study their potential therapeutic targets and methods and have made interesting findings. For example, Ren et al. reported that treatment of CAFs with AC1MMYR2, a small molecule inhibitor of miR-21, can suppress tumor growth and metastasis and improve chemosensitivity [169]. However, in practical applications, miRNA-targeted therapies do not simply promote or inhibit the expression of miRNAs. We previously mentioned that one miRNA can regulate the expression of multiple genes; thus, when a drug acts on a target miRNA, off-target effects inevitably occur. Hence, identifying the best miRNA targets in different kinds of tumors is highly challenging. Lai et al. proposed the use of cooperating miRNAs to treat tumors, aiming to minimize toxicity and reduce off-target effects, but high-quality clinical trials are needed to confirm the feasibility of this strategy [170]. In addition, because RNA molecules are unstable, it is necessary to modify the molecules or develop an effective delivery system for miRNA-targeted therapy; moreover, the safety of these methods should be considered [171]. Indeed, one clinical trial (NCT01829971) was terminated due to immune-related toxicity in several patients. Therefore, miRNA-targeted therapy needs further exploration.

#### Targeting exosomes

Tumor-promoting ncRNAs loaded in exosomes are vital for tumor progression after being taken up by tumor cells or stromal cells. Therefore, many studies are devoted to blocking the effect of exosomes from many directions, such as suppressing their formation, release and uptake, to treat cancer [172]. Marleau et al. developed a system called adaptive dialysis-like affinity platform technology that aims to treat tumors by capturing and removing tumor-related exosomes from the circulation [173]. On the other hand, as mentioned earlier, an effective and safe delivery system is required in RNA-targeted therapy, and exosomes meet this need. Currently, a key direction in exosome research is isolating exosomes, combining them with ncRNAs that act as tumor suppressors, and then effectively transferring them to tumor tissues to obtain therapeutic effects. This direction is also being pursued in research on CAFs. Wang et al. found that the expression of miR-335-5p was downregulated in HCC cells and CAFs, while the invasive ability of HCC cells in an in vitro model was decreased after treatment with exosomal miR-335-5p. Further studies using animal models have shown that exosomal miR-335-5p can inhibit tumor growth in vivo by reducing proliferation and increasing apoptosis [174]. Another study by the same team also demonstrated that exogenous exosomal miR-195 can inhibit the growth of cholangiocarcinoma in vivo and prolong survival [175]. These studies indicate the potential prospects of exosome replacement therapy.

## LncRNAs in CAFs

### Biosynthesis and mechanisms of lncRNAs

LncRNAs are a class of RNA molecules with sequences longer than 200 nt that includes mainly long intergenic noncoding RNAs (lincRNAs) and natural antisense transcripts (NATs) of protein-coding genes [176, 177]. Like mRNAs, most lncRNAs are transcribed by RNA polymerase II and have a 5′ m^7^G cap and 3′ poly(A) tail. However, there are other differences; for example, lncRNAs have cis-regulation ability but lack translated ORFs and coding ability [178]. Importantly, the overall family of lncRNAs also includes unconventional lncRNAs derived from primary RNA polymerase II-derived transcripts, and the 3′ ends of these lncRNAs are processed via a unique mechanism. For instance, two types of unconventional lncRNAs, snoRNA-ended lncRNAs (sno-lncRNAs) and 5′-snoRNA-ended and 3′-polyadenylated lncRNAs (SPAs), enhance the structural stability of RNA molecules by capping one or both of their ends via small nucleolar ribonucleoproteins (snoRNPs) [179]. Due to the operational definition of lncRNAs, some researchers divided them into various subsets according to different characteristics, as reviewed in Ref.[180].

LncRNAs have more diverse functions than miRNAs, and these functions cannot be simply inferred from their sequence or structure. Chromatin modification is an important function of lncRNAs. LncRNAs can interact with chromatin-modifying complexes and mediate epigenetic changes by recruiting these complexes to specific genomic loci [181]. Another function of lncRNAs is transcriptional regulation. LncRNAs can transcriptionally regulate the expression of genes in cis or target distant transcriptional activators or inhibitors, a process called trans-action [182]. In addition, lncRNAs can regulate the activities of RNA polymerase II and transcription factors through different mechanisms [183]. LncRNAs have also been found to bind directly to DNA to alter gene expression via the formation of R-loops or RNA–DNA triplex structures [184, 185]. However, lncRNAs also play a pivotal role in posttranscriptional regulation. Recently, many lncRNAs have been identified to regulate gene expression at the posttranscriptional level by changing the function and integrity of nuclear bodies [14]. Additionally, lncRNAs may mediate specific interactions associated with multiple steps of posttranscriptional mRNA processing, such as splicing, transport and degradation. Interestingly, some nuclear-localized lncRNAs have been found to be related to ribosomes, which proves that lncRNAs may be involved in translational regulation [186].

### LncRNAs in the formation and activation of CAFs

LncRNAs contribute to the formation and activation of CAFs. A new pan-cancer integration strategy was applied to classify lncRNAs into 16 modules according to different functional signatures. Twelve lncRNAs involved in one of the modules were found to be closely related to the formation of CAFs. The authors carried out siRNA knockdown experiments and observed that the transformation of NFs to CAFs was decreased, confirming this correlation [187]. In another study, a lncRNA significantly upregulated in CAFs was identified from oral squamous cell carcinoma (OSCC) by RNA sequencing; this lncRNA was found to promote the transformation of NFs to CAFs and affect the proliferation of cancer cells by increasing the IL-33 level [188]. Furthermore, Hu et al. found that the lncRNA Gm26809 in exosomes secreted from melanoma cells was taken up by NFs and reprogrammed them into CAFs, thus facilitating the proliferation and migration of melanoma cells [189].

### LncRNAs in the effect of CAFs on cancer cells

**Fig. 3:**
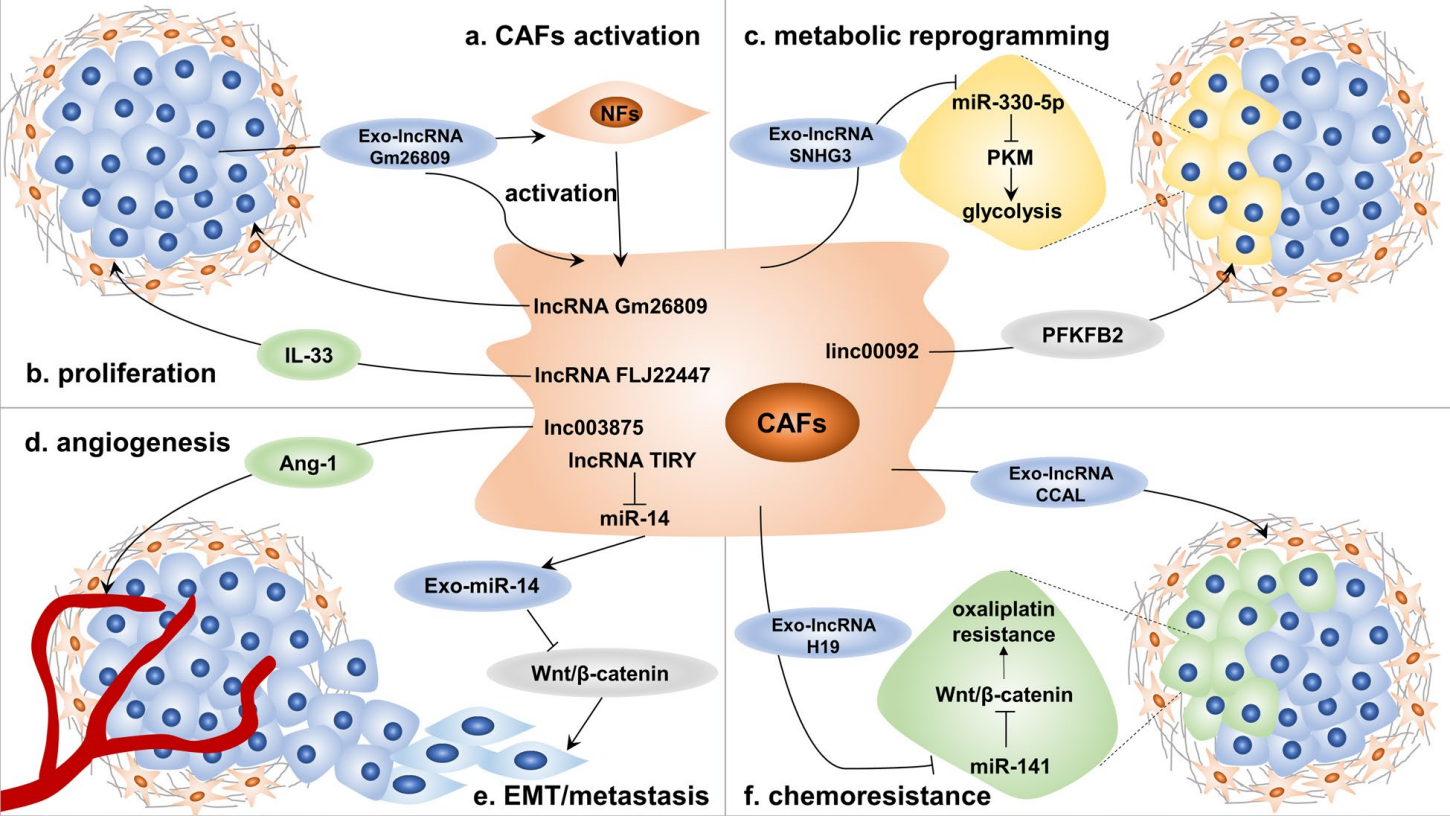
Long noncoding RNAs (lncRNAs) in cancer-associated fibroblasts (CAFs). LncRNAs mediate the crosstalk between tumor cells and CAFs through a variety of mechanisms: **a** exosomal lncRNAs from tumor cells are taken up by normal fibroblasts (NFs) to promote their activation into CAFs; **b** lncRNA dysregulation in CAFs leads to increases in cytokines such as IL-33, which can enhance the proliferation of tumor cells; **c** both lncRNA dysregulation and exosomal lncRNAs from CAFs target different key metabolic enzymes, such as pyruvate kinase M1/M2 (PKM) and 6-phosphofructo-2-kinase/fructose-2, 6-biphosphatase 2 (PFKFB2), to mediate metabolic reprogramming of tumor cells; **d** lncRNA dysregulation increases the secretion of proangiogenic factors such as angiopoietin-1 (Ang-1), thus promoting tumor angiogenesis; **e** lncRNAs act as microRNA (miRNA) sponges to reduce the secretion of exosomal miRNAs, resulting in epithelial-to-mesenchymal transition (EMT) of tumor cells and the acquisition of metastatic ability; and **f** tumor cells acquire chemoresistance by taking up exosomal lncRNAs

With the continuous emphasis on the role of lncRNAs in the TME, researchers have begun to consider the function of lncRNAs in CAFs, as well as the effects of lncRNA dysregulation and exosomal lncRNAs derived from CAFs on cancer cells (Fig. 3) [190, 191].

#### Metastasis and metabolic reprogramming

The effect of lncRNAs in CAFs on cancer metastasis has been verified in many studies. Vafaee et al. analyzed the RNA expression of CAFs and NFs in ovarian cancer and identified 39 differentially expressed lncRNAs, some of which can predict cancer metastasis [192]. A recent study demonstrated that lnc003875 induces cancer metastasis by promoting angiogenesis [193]. In placental site trophoblastic tumors, lnc003875 upregulates the expression of early growth response 1 (EGR1), which promotes angiopoietin-1 (Ang-1) secretion by CAFs to enhance angiogenesis. As mentioned in the previous section, EMT is a crucial mechanism in cancer metastasis. The lncRNA TIRY can decrease the level of exosomal miR-14 derived from CAFs in OSCC as a miRNA sponge. MiR-14 inhibits the Wnt/β-catenin signaling pathway by targeting *Wnt3A*, thus inhibiting EMT of cancer cells. However, the lncRNA TIRY activates this pathway and finally induces the invasion and metastasis of OSCC cells [194]. Importantly, in ovarian cancer, linc00092 maintains the local supportive function of CAFs by binding to 6-phosphofructo-2-kinase/fructose-2,6-biphosphatase 2 (PFKFB2) to promote glycolysis in cancer cells, thus contributing to cancer metastasis [195]. Another study revealed that exosomal lncRNA SNHG3 secreted from CAFs acts as a miRNA sponge to decrease the level of miR-330-5p in cancer cells, which in turn increases the expression of pyruvate kinase M1/M2 (PKM), one of the main rate-limiting enzymes in glycolysis [196]. These results indicate that the substantial effect of lncRNAs on metabolic regulation is also reflected in the crosstalk between CAFs and cancer cells.

#### Chemoresistance

LncRNAs in CAFs also play an important role in chemoresistance. Enrichment of the lncRNA H19 in exosomes secreted from CAFs can promote the stemness of colorectal CSCs and the oxaliplatin resistance of colorectal cancer cells in vitro and in vivo [197]. Interestingly, H19 can also act as a sponge of miR-141 to regulate the expression of β-catenin, and the Wnt/β-catenin pathway is essential for the maintenance of CSCs and chemoresistance of cancer cells. Similarly, another study confirmed that exosomal colorectal cancer-associated lncRNA (CCAL) derived from CAFs upregulates the expression of β-catenin by interacting with the mRNA-stabilizing protein HuR, which promotes the oxaliplatin resistance of colorectal cancer cells [198].

### LncRNAs in CAFs as biomarkers for cancer prognosis

A recent study illustrated the prognostic value of lncRNAs in CAFs. This study identified 10 differentially expressed lncRNAs associated with poorer overall survival in ovarian cancer CAFs [199]. In particular, 9 of these lncRNAs were differentially expressed only in CAFs, not in tumor cells, which reveals the vital functions of lncRNAs in CAFs. Importantly, the high expression of MIR155HG, one of the identified lncRNAs, is associated with higher immune cell infiltration, which may be a reason for its impact on patient prognosis. In addition, as shown in Table 3, the lncRNA FLJ22447 can also be used as a prognostic factor in OSCC [188].

### Potential therapeutic approaches for targeting lncRNAs

LncRNAs are transcripts similar to mRNAs; thus, many molecules that act on mRNAs, such as antisense oligonucleotides (ASOs), siRNAs, small molecule antagonists, and ribozymes, can also be used to target lncRNAs [200]. ASOs are chemically modified, synthetic single-stranded oligonucleotides that can bind to complementary sequences in RNA molecules, resulting in RNase H-dependent degradation of the target lncRNAs [201]. RNA interference is another important means of targeting lncRNAs. SiRNAs bind to lncRNAs and induce the formation and activation of the RISC, which finally leads to degradation of the lncRNAs. Importantly, lncRNAs can exert a marked effect by loading into exosomes. Therefore, targeting exosomes (which were introduced earlier) is also a potential therapeutic approach. Although there are many strategies for lncRNA-targeted therapy, because studies on lncRNAs in CAFs are still in the preliminary stage, we need to further understand their function and mechanism to develop more effective treatments.

## Conclusions

The importance of the TME, as a key regulator of tumor progression, has received increasing attention. A deeper understanding of CAFs, a kind of stromal cell that is the main component of the TME, is very helpful for us to further clarify the mechanisms by which the TME contributes to tumor progression. CAFs are a heterogeneous population of cells of multiple origins that mainly promote the development of tumors. However, some recent studies have shown that they can also have tumor-suppressive effects. Although some studies have reported that gene mutations are present in CAFs, such mutations are generally believed to be uncommon. Therefore, the phenotypic and functional changes in CAFs are regulated mainly by other factors, among which ncRNAs play a crucial role. As reviewed herein, miRNAs participate in the formation and activation of CAFs and modulate their metabolic reprogramming, which is one of the important characteristics that distinguishes CAFs from NFs and is a prerequisite for the role of CAFs. This process can be accomplished through miRNA dysregulation in CAFs or mediated by exosomal miRNAs secreted from tumor cells. Subsequently, through miRNAs, activated CAFs can regulate numerous biological functions in tumor cells, including proliferation, stemness, metabolism, angiogenesis, invasion, metastasis and the acquisition of therapeutic resistance. These regulatory effects can be exerted through miRNA dysregulation in CAFs, which leads to the transformation of the matrix-regulatory function of CAFs and the secretion of related molecules to regulate tumor cells. Similarly, through secreted exosomes, exosomal miRNAs can be transferred to tumor cells and act directly on the corresponding targets to regulate a series of biological characteristics. It can be seen that miRNAs are involved in almost all aspects of communication between CAFs and tumor cells. Moreover, studies on lncRNAs in CAFs are being carried out gradually and have shown the expected results, which reveal that lncRNAs also contribute to tumor progression via many mechanisms. Interestingly, some studies have shown that lncRNAs act as miRNA sponges to regulate the level of miRNAs, linking the study of lncRNAs with the study of miRNAs. However, most of the studies mentioned in our review have focused on the mechanism of a single signaling pathway mediated by a single ncRNA, and further research on the complex regulatory networks in the TME is lacking. Therefore, the conclusions of our studies may be one-sided and address only part of a large and complex network. As research on ncRNAs intensifies, we should conduct more in-depth studies on the regulatory networks mediated by ncRNAs to identify both the overall function of a single ncRNA and the synergistic functions of multiple ncRNAs and to validate the driving factors and causal relationships. This knowledge will help us to identify the optimal single or multiple ncRNA targets in order to design more effective and safer treatments. Last but not least, the in-depth study of ncRNAs in CAFs can enable us to further understand the nature and biological characteristics of CAFs and may facilitate the identification of additional treatments targeting CAFs, rather than just treatments targeting ncRNAs.

## Data Availability

Not applicable.

## Abbreviations

3′ UTR: 3′ Untranslated region
ALOX15: Arachidonate lipoxygenase 15
Ang-1: Angiopoietin-1
apCAFs: Antigen-presenting CAFs
ASOs: Antisense oligonucleotides
BCL2: B-cell lymphoma 2
BMP: Bone morphogenetic protein
CAFs: Cancer-associated fibroblasts
CCAL: Colorectal cancer-associated lncRNA
CDK2: Cyclin-dependent kinase 2
circRNAs: Circular RNAs
CSCs: Cancer stem cells
CXCL: C-X-C motif chemokine ligand
CXCR: C-X-C motif chemokine receptor
DGCR8: DiGeorge critical region 8
ECM: Extracellular matrix
EGF: Epidermal growth factor
EGR1: Early growth response 1
EMT: Epithelial-to-mesenchymal transition
EndoMT: Endothelial-to-mesenchymal transition
FAP: Fibroblast activation protein
FGF2: Fibroblast growth factor 2
HCC: Hepatocellular carcinoma
HSCs: Hepatic stellate cells
HUVECs: Human umbilical vein endothelial cells
iCAFs: Inflammatory CAFs
IL: Interleukin
lincRNAs: Long intergenic noncoding RNAs
lncRNAs: Long noncoding RNAs
MDSCs: Myeloid-derived suppressor cells
miRNAs: MicroRNAs
MMP: Matrix metalloproteinase
MSCs: Mesenchymal stem cells
MTOC: Microtubule-organizing center
myCAFs: Myofibroblastic CAFs
NATs: Natural antisense transcripts
ncRNAs: Noncoding RNAs
NFs: Normal fibroblasts
ORFs: Open reading frames
OS: Overall survival
OSCC: Oral squamous cell carcinoma
PAIs: Plasminogen activator inhibitors
PAR-4: Protease-activated receptor 4
PDAC: Pancreatic ductal adenocarcinoma
PDGF: Platelet-derived growth factor
PEDF: Pigment epithelium-derived factor
PFKFB2: 6-Phosphofructo-2-kinase/fructose-2,6-biphosphatase 2
piRNAs: Piwi-interacting RNAs
PKM: Pyruvate kinase M1/M2
PSCs: Pancreatic stellate cells
RBPs: RNA-binding proteins
RISC: RNA-induced silencing complex
ROS: Reactive oxygen species
SCC: Squamous cell carcinoma
SDF-1: Stromal cell-derived factor 1
SHH: Sonic hedgehog
siRNAs: Small interfering RNAs
sncRNAs: Small noncoding RNAs
sno-lncRNAs: SnoRNA-ended lncRNAs
snoRNPs: SnoRNA-protein complexes
SOCS1: Suppressor of cytokine signaling 1
SPAs: 5′-SnoRNA-ended and 3′-polyadenylated lncRNAs
TCA: Tricarboxylic acid
TGF-β: Transforming growth factor-β
TME: Tumor microenvironment
VEGF: Vascular endothelial growth factor
α-SMA: α-Smooth muscle actin
